# Morphological, molecular, and biological characterization of bulb rot pathogens in stored Lanzhou lily and the *in vitro* antifungal efficacy of three plant essential oils

**DOI:** 10.3389/fmicb.2024.1307966

**Published:** 2024-04-11

**Authors:** Chaoqun Liu, Yinquan Wang, Ling Jin, Yan Wang, Dongling Liu

**Affiliations:** ^1^College of Pharmacy, Gansu University of Chinese Medicine, Lanzhou, China; ^2^Northwest Collaborative Innovation Center for Traditional Chinese Medicine Co-Constructed by Gansu Province and MOE of PRC, Lanzhou, China

**Keywords:** *Lilium davidii* var. *willmottiae* (E. H. Wilson) Raffill, bulb rot disease, *Fusarium oxysporum*, biological characteristics, anti-fungal effect, plant essential oils

## Abstract

Lanzhou lily (*Lilium davidii* var. *willmottiae*) is an exclusive sweet lily variety indigenous to China, which is susceptible to bulbous rot caused by fungal infection during storage. This experiment tests the pathogenicity of the pure culture isolated from the diseased tissue was confirmed in accordance with Koch's postulates, and the pathomycetes were identified based on their morphological and molecular characteristics. Furthermore, the biological characteristics of the pathogens were investigated, followed by an evaluation of the antifungal effects of three plant essential oils against them. The results showed that two strains of fungi were isolated from Lanzhou lily rot, which were identified as *Fusarium oxysporum* Schl. and *Aspergillus sydowii* (Bain. Et sart.). In addition, the pathogenicity of these two strains of fungi was demonstrated that only *F. oxysporum* induced rot with similar symptoms during the post-harvest storage period. The biological characteristics of *F. oxysporum* indicated the potato maltose agar and lily dextrose agar were identified as the most suitable media. Sucrose was determined to be the optimal carbon source, while ammonium nitrate was found to be the best nitrogen source for the growth of *F. oxysporum*. Mycelial growth and sporulation of *F. oxysporum* occurred at an optimum pH value of 6. Total darkness facilitated mycelial growth and conidial germination. The ideal temperature for growth was found to be 28°C, while relative humidity did not significantly impact mycelial growth; however, a relative humidity of 55% was most favorable for spore production. Among the three essential oils tested, cinnamon essential oil displayed superior antifungal efficacy against *F. oxysporum*, whereas angelica essential oil and tea tree essential oil also exhibited moderate inhibitory effects against this pathogen. This research provides valuable theoretical insights for disease control during the storage and transportation of Lanzhou lily.

## Introduction

Over 200 species belonging to the *Lilium L*. family can be found worldwide, and China serves as the primary geography for *Lilium* as 55 *Lilium* species are grown here (Kong et al., 2017). Lanzhou lily (*Lilium davidii* var. *willmottiae*) represents a variety of *L. davidii* within the *Liliaceae* family (Hu et al., 2020). It is just an exclusive sweet lily variety native to China characterized by its white color and sweet taste. Moreover, it contains significant amounts of natural phospholipids, alkaline elements, pectin, abundant cellulose, and plant protein. Lanzhou lily possesses various medicinal effects such as fortifying the spleen and nourishing the stomach while also acting as an anti-aging agent and offering protection against atherosclerosis. Additionally, it exhibits numerous bioactivities as an antioxidant and also bestows antitumor effects, glucose-lowering benefits, and immunomodulatory effects (Gao D. et al., 2022). Therefore, Lanzhou lily has been utilized for both medicinal and culinary purposes for more than 400 years in China. Lanzhou, situated in the northwestern region of the Chinese Loess Plateau in Gansu, boasts a dry climate and high altitude with significant temperature fluctuations between day and night. These conditions provide an ideal environment for the accumulation of nutritional and bioactive substances in plants, therefore conducive to the cultivation of high-value economic vegetables. As such, it has been recognized by the General Administration of Quality Supervision, Inspection, and Quarantine of the People's Republic of China as a geographical indication protected product (Li et al., 2020). The cultivation area of Lanzhou lily has currently expanded to 13,000 hectares, yielding approximately 80,000 tons. This industry serves as the cornerstone for farmers' prosperity in the primary production regions.

Lanzhou lily is vulnerable to mechanical damages during harvesting, storage, and transportation and pathogenic fungi infiltrate through wounds on its succulent roots or bulbs, resulting in bulb discoloration and decay (Janisiewicz and Conway, 2010). Statistical data indicates an estimated annual post-harvest loss of Lanzhou lily ranging from 20 to 25%. Fungal infection is the major factor contributing to changes in safety and sensory attributes, degradation of functional compounds, production of odors, and reduction in edible quality during the storage of Lanzhou lily (Sanches-Silva et al., 2014).

The occurrence of bulb rot disease (BRD) is commonly associated with bulb damage, a humid storage environment, and poor ventilation. Typically, it manifests as brown sunken spots on the outer bulb, with the central part of the spot exhibiting rotting, which spreads to the surrounding area. The affected tissue continues to decay until the entire bulb undergoes dry rotting (Bian, 2016; Jiao et al., 2021). BRD and wilt in the *Lilium* oriental hybrid cultivar “Sorbonne” are caused by *Fusarium tricinctum* (Corda) Sacc., *Fusarium oxysporum*, and *Fusarium solani* (Li et al., 2013; Cao et al., 2018). In addition, *F. tricinctum* and *Aspergillus flavus* not only cause wilt disease in Chinese ornamental lilies but also affect edible lilies in Lanzhou (Shang et al., 2014; Gong et al., 2015). In binucleate bulbs afflicted by BRD in Israel, fungal pathogens such as *Rhizoctonia solani* Kühn, *Pythium oligandrum* Drechsler, *Fusarium proliferatum*, and *F. oxysporum* have been identified. Among them, *R*. AG-A, *P. oligandrum*, and *F. proliferatum* can cause significant damage to bulbs (Sara et al., 2014; Xing et al., 2022). *F. oxysporum* f.sp. Lilii has been found responsible for medical lily bulb rot in Hunan, Jiangxi, Zhejiang, and Beijing in China. *F. solani* causes BRD in Zhejiang and Jiangxi, while *F. proliferatum* and *F. commune* are the agents causing this disease in Jiangxi. *Curvularia pseudobrachyspora* has been reported as a causal agent for *Lilium brownii* var. *viridulum* BRD in Jiangxi (Zeng et al., 2020; Jiao et al., 2021). Obviously, a majority of these pathogens were isolated from the decaying bulbs of ornamental lilies, with limited research being conducted on BRD in Lanzhou lily bulbs during storage.

The use of fungicides to prevent or treat plant phytopathogenic fungi has given rise to the development of resistant strains of fungal pathogens and has consequently engendered a plethora of health hazards, including an escalated risk of cancer and environmental contamination (Panjehkeh and Jahani Hossein-Abadi, 2011). With increasing focus on food safety, there is a growing demand for broad-spectrum, efficient, environmentally friendly, and safe food preservatives (Wei et al., 2018). Natural plant ingredients possessing antifungal properties are expected to serve as alternatives to chemical fungicides (Zhu et al., 2019). Plant essential oils represent a class of plant secondary metabolites that exhibit diverse biological activities and can be abundantly found in the roots, stems, leaves, flowers, and fruits of plants (Maffei, 2010). Approximately 60 plant families encompass species that produce essential oils. The Apiaceae, Asteraceae, Lamiaceae, Lauraceae, and Camelliaceae families are particularly significant due to their essential oils possessing anti-fungal and anti-bacterial effects and other properties (Vigan, 2010; Hammer and Carson, 2011). However, there is currently no available literature documenting the utilization of cinnamon essential oil, tea tree essential oil, and angelica essential oil for the prevention and control of storage diseases in Lanzhou lily bulbs.

The objective of this research was to isolate and identify the pathogen that causes BRD in Lanzhou lily and investigate its morphological, molecular, and biological characteristics. Additionally, we assessed the potential of antifungal plant essential oils against BRD *in vitro*. This research serves as a valuable theoretical reference for disease control during the storage and transportation of Lanzhou lily bulbs.

## Materials and methods

### Sample collection

Lanzhou lily bulb rot samples were collected from Yuanjiawan Village, Xiguoyuan, Qilihe District, Lanzhou, China (36.065915°N, 103.785866°E) in late October in 2022 and stored at −4°C in the bottom fresh-keeping refrigerator. The bulb rot collected had diseased plants with typical symptoms of BRD.

#### Isolation and purification of fungus

Approximately 50 rotten bulbs with typical BRD symptoms during storage were collected and pathogenic fungi were isolated by tissue separation. The bulb rot was washed with water, cut 4 × 4 mm in size at the place where diseased, and healthy tissues were placed in the ultraclean workbench. The surface of the diseased bulb was disinfected with 75% absolute alcohol for 30 s, washed three times with sterilized water, disinfected with 2% (v/v) sodium hypochlorite solution for 2.5 min, rinsed three times with sterile water, placed on potato dextrose agar (PDA) medium, and incubated at 28°C for 3–5 days. The edge of the colony with a hole punch was picked up and placed on the PDA medium for purification. After the mycelium grows out, the purified colony was then isolated and cultured for a single spore.

#### Pathogenicity test

To confirm the pathogenicity of pure culture isolated from the lily samples with BRD, Koch's postulates were performed (Gao D. et al., 2022; Gao Z. Y. et al., 2022). In this experiment, conidia obtained from the 1-week-old culture medium that carried with fungal isolate were used. First, the surface of 35 asymptomatic lily bulb samples was disinfected with 75% alcohol, washed with sterile distilled water, then disinfected by sodium hypochlorite solution (2%, v/v) for 2.5 min. Subsequently, sterile distilled water was used to rinse them to complete surface disinfection. The bulbs were air-dried for 10 min at room temperature (25 ± 2°C; De Oliveira et al., 2014). The sterilized inoculum was used to puncture the healthy bulb. The fungi cake with PDA culture medium were pasted on the hole as the injured inoculation, and the fungi cake with PDA culture medium were pasted on the bulb slice without puncture as the uninjured inoculation. The healthy bulb slice was treated as the control and five bulbs per group were placed. This process was repeated seven times. The bulbs were put into the culture dish for moisture culture. All bulbs were incubated at 28°C under light/dark for 12 h and 60–70% relative humidity after inoculation. After 15 days, the disease incidence was recorded to determine the pathogenicity of the pathogen to the bulbs. The aforementioned single-spore isolation and purification were used to re-isolate fungi from each group of lesions observed on the inoculated corms to prove Koch's hypothesis.

#### Pathogen identification

##### Morphological identification

The appearance of colonies and morphology of the fungal isolates were surveyed after placing them in a biochemical incubator at light/dark for 12 h at 28°C for a week. The representative isolate was used for microscopic observations. The morphological characteristics of the hyphae and spores of the pathogenic fungi were observed and photographed with a fluorescence microscope system (Leica DM4000B LED, Germany). The size and morphological information related to the characteristics of the strain (e.g., hypha, macroconidium, and microconidium) were measured with all 50–60 numbers of each structure.

#### Molecular identification

##### DNA extraction, amplification, and sequencing

The total genomic DNA of a 5-day-old fungal isolate cultivated on a PDA medium at 28°C was extracted using a fungal/bacterial DNA kit^TM^ (Favorgen, Taiwan). Polymerase chain reaction (PCR) amplification of ITS, Tef-1, Cam, PRB1, PRB2, TUB2, BenA, Mcm7, and Tsr1 was performed using the primer pairs according to the methods described earlier (Bian et al., 2022; Sklenár et al., 2022; Nuangmek et al., 2023). The relevant gene sequences are listed in Table 1 (Wang et al., 2022). The amplification of nine genes was completed in an independent PCR reaction. The program employed for amplification is as follows: one initial step of 3 min at 95°C; the second step of 35 cycles for 30 s at 95°C, next, annealing step for 50 s at 60°C and 30 s at 59°C or 1 min at 52°C; finally, the extension step lasting 10 min at 72°C. The PCR products were directly sequenced followed by final purification. Sequencing reactions were carried out and the aforementioned PCR primers were employed in the ABI Prism 3130 genetic analyzer (Applied Biosystems) according to the manufacturer's instructions.

**Table 1:** Primers identified in the PCR amplification of the seven loci.

**Locus**	**Primer**	**Sequence of primer (5^′^-3^′^)**
ITS	ITS5	GGAAGTAAAAGTCGTAACAAGG
	ITS4	TCCTCCGCTTATTGATATGC
Tef1	EF1	ATGGGTAAGGARGACAAGAC
	EF2	GGARGTACCAGTSATCATG
Ccam	CL1	GARTWCAAGGAGGCCTTCTC
	CL2A	TTTTTGCATCATGAGTTGGAC
RPB1	RPB1-Fa	CAYAARGARTCYATGATGGGWC
	RPB1-G2R	GTCATYTGDGTDGCDGGYTCDCC
RPB2	RPB2-5f2	GGGGWGAYCAGAAGAAGGC
	RPB2-11ar	GCRTGGATCTTRTCRTCSACC
Tub2	T1	AACATGCGTGAGATTGTAAGT
	T2	TAGTGACCCTTGGCCCAGTTG

#### Phylogenetic analyses

The genes such as Cam, Tef-1, PRB1, PRB2, TUB2, BenA, Mcm7, and Tsr1 obtained from the sequences were compared with those from the NCBI for similarity analysis via http://blast.ncbi.nlm.nih.gov (accessed on January 13, 2024). Multiple proofreads were arranged by edgar (Nuangmek et al., 2023) and alignment was manually adjusted if necessary. The obtained data about Tef-1, Cam, PRB1, PRB2, TUB2, BenA, Mcm7, and Tsr1 was used for multi-gene phylogenetic analysis (Sklenár et al., 2022; Nuangmek et al., 2023). Phylogenetic analysis was performed using maximum likelihood (ML) and Bayesian inference (BI) methods. Nucleotide substituted Gtrcat model on RAxML-HPC2 version 8.2.12 (Nuangmek et al., 2023) on the Cipres internet portal (Miller et al., 2010) was employed. The best pattern for nucleotide substitution was derived using the J-Modeltest V.2.3 (Darriba et al., 2012) on the basis of the designated method (Ronquist et al., 2012). The BI analysis was conducted following the protocol of Wang et al. (2019). Using MrBayes V. 3.2.6 (Ronquist et al., 2012) in combinatorial analysis, each locus is optimized into a partition. All characters have equal weights, and blank spaces are considered missing data. The phylogenetic trees were visualized using FigTree v1.4.0 (Rambaut, 2019).

#### Influence of different culture media on mycelium growth and sporulation quantity

A 1-week-old fungi block with a diameter of ~6 mm was placed in the middle of different types of tested media, such as PDA medium, potato maltose agar (PMA) medium, lily agar (LA) medium and lily dextrose agar (LDA) medium. Then the culture was developed at 28°C for 3–5 days under light/dark for 12 h. The diameter of mycelium is usually measured using the cross-shaped method on the 3rd day. The blood cell counting plate method is used to measure the spore yield 5 days later.

#### Utilization of different carbon and nitrogen sources

As mycelium grows from carbon and nitrogen sources, 7-day-old colonies were placed in the pivot of the culture. The growth of mycelium and sporulation yield of the pathogen from various nitrogen sources and carbon sources were calculated by Cha's solid (PTT) medium (Li et al., 2021). To sum up, two different types of carbon sources were used, namely, sucrose (ZT) and fructose (GT). No carbon source was treated as the control (PTT) group. At the same time, the method for setting nitrogen source groups were the same as above using ammonium nitrate (XSA) and ammonium sulfate (LSA). No nitrogen source was treated as the control (PTT). The experiments were repeated three times for each group and then mycelial development was observed at 28°C for 3–5 days under light/dark for 12 h. Mycelial growth and spore production were measured on the basis of the above method.

#### Effect of temperature and relative humidity (RH) on mycelium growth and sporulation quantity

The effect of temperature and RH on the growth of mycelium and sporulation quantity was tested on PDA medium, by incubating the culture at temperatures of 4, 15, 28, and 37°C and at relative humidity ranging from 55 to 85% at 15% intervals. A mature colony of 6 mm in diameter was placed at the center of the medium, which was then incubated at different temperatures and RH. The growth of mycelial was tested on the 3rd day. The sporulation quantity under different temperatures and relative humidity was determined.

#### Influence of pH on mycelium growth and sporulation quantity

To study the influence of pH on mycelium growth and sporulation quantity, the culture was incubated at pH ranging from 5 to 9. A mature colony, which contained the isolated strains, was placed in PDA medium with a diameter about 6 mm and then incubated at 28°C. The spore yield was measured by blood cell counting plate at 7 days, and colony growth was measured by cross method as an indicator of mycelial growth.

#### Influence of photoperiod on mycelium growth and sporulation quantity

To understand the influence of illumination by light, the PDA medium was placed in incubators at 28°C under three light conditions of full light, full darkness, and light/dark for 12 h, respectively, which was then inoculated by pathogenic fungi blocks at 6 mm centrally. Each process was repeated three times. The mycelial growth and spore production were measured on the basis of the abovementioned method.

#### In vitro antifungal susceptibility of plant essential oils on mycelial growth

The growth of mycelium reflects the antifungal susceptibility of fungicides (Xin et al., 2020) to a certain degree. The various of anti-fungal agents were blended with the liquid culture of PDA medium at different concentrations in a moderate temperature. A comparable sterile water and carbendazim were added to the PDA medium, which served as the control group and positive control group, respectively. Meanwhile, PDA medium was taken as a blank control group. Afterward, a colony with fungi was placed in the blank control group, cultured at the conditions of 28°C and 55% RH for 7 days under light/dark for 12 h. Next the diameter of the mycelial growth (cm) was measured by a ruler. The employed essential oils were cinnamon essential oils (CEOs), tea tree essential oils (TEOs), and angelica essential oils (AEOs), which extracted from the stems and leaves of cinnamon, tea trees, and *Angelica sinensis* and carbendazim purchased from Sichuan Runer Technology Co., Ltd. (Pesticide registration number: PD85150-35).

#### Data analysis

SPSS 22.0 statistical software was utilized for one-way analysis of variance (ANOVA), Data analysis and plotting were performed using GraphPad Prism 8.0.2 and Duncan's new multiple range method was employed to test the significance of differences among treatments. The level of significance was set at *P* = 0.05.

## Results

### Pathogen identification

#### Morphological observations

**Figure 1:**
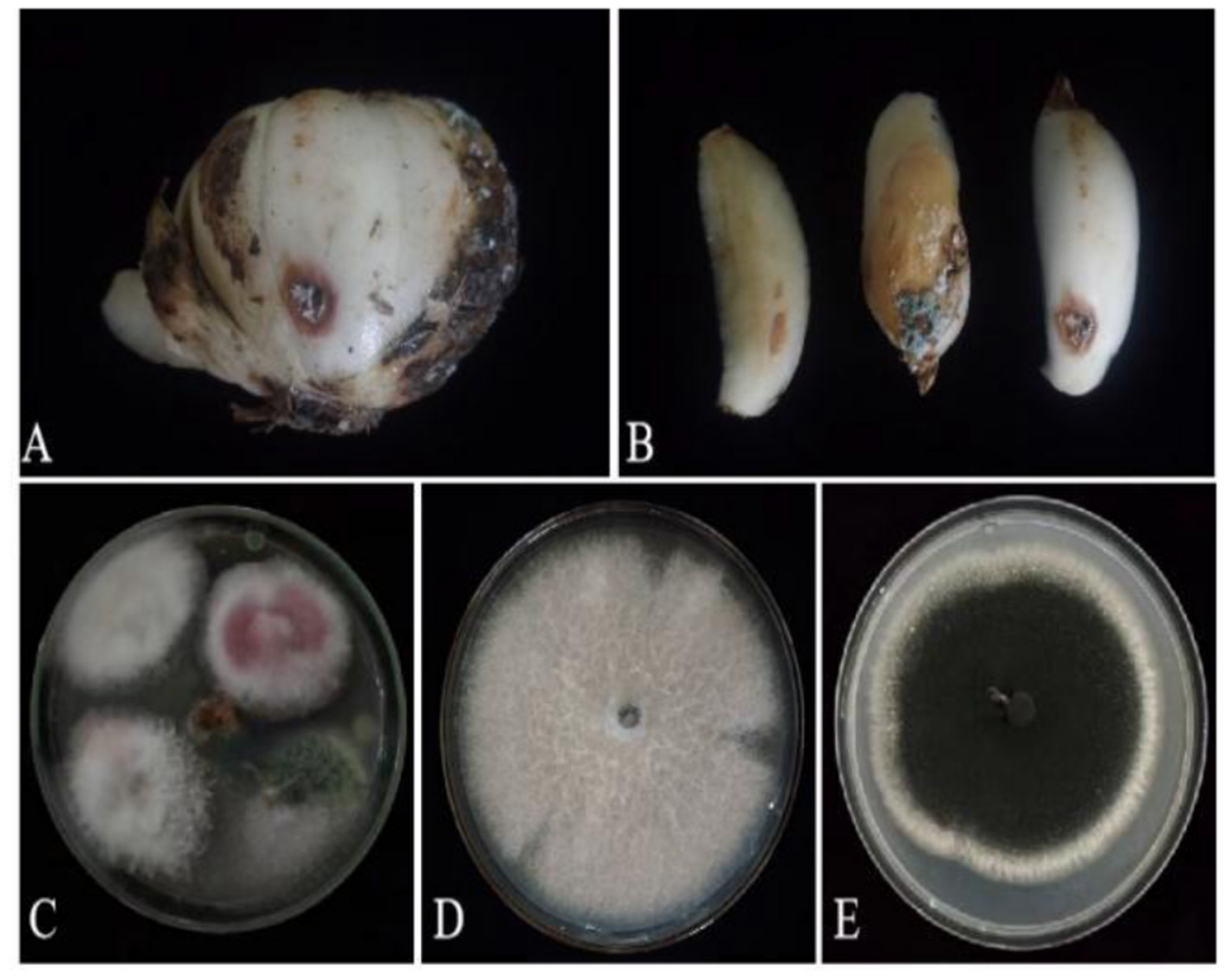
Lily samples with BRD **(A, B)**, separators of lily samples **(C)**, and pathogens of lily separators **(D, E)**.

**Figure 2:**
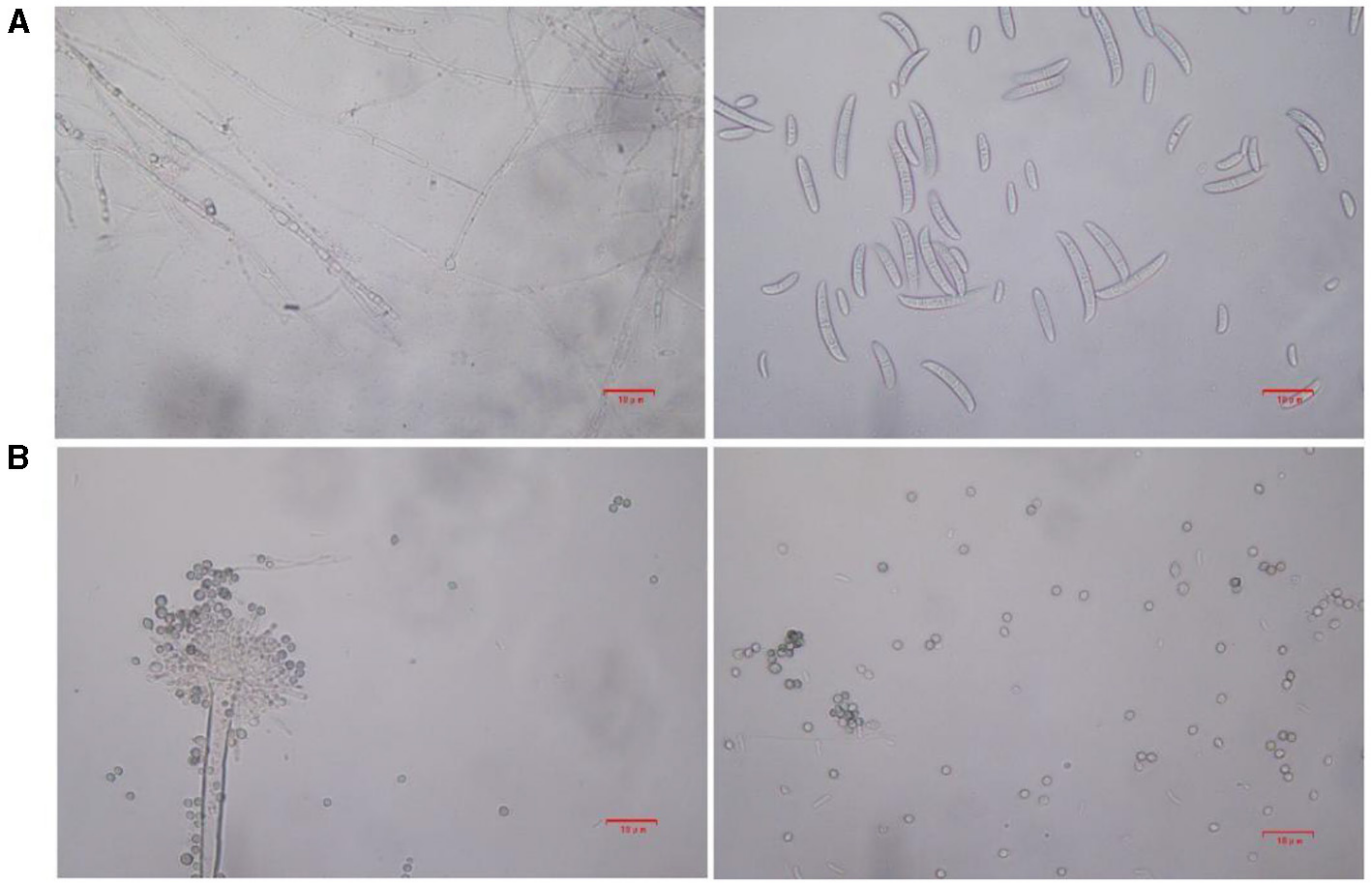
The microstructure of strains 201 and 202 in the logarithmic phase. **(A)** The effect of strain 201 on separating mycelium and spore under 400×, scale bars: D–G = 10 μm. **(B)** The effect of strain 202 on separating mycelium and spore under 400X, scale bars: D–G = 10 μm.

Based on lily samples with BRD symptoms (Figures 1A, B), we used tissue separation method to obtain isolated strains (Figure 1C). The two strains (hereafter referred to as strains 201 and 202) were obtained through tissue isolation, and two colonies of each isolate were placed on PDA medium at a temperature of 28°C for 1 week. Strain 201 exhibited a white color with a regular circular shape, accompanied by the exudate in the early stage. The central part appeared flocculent, dense, and velvety, gradually transitioning from colorless to light yellow and becoming fluffy after 7 days. The phenomenon of light yellow pigmentation can be seen on the back surface of the PDA medium (Figure 1D). On the other hand, strain 202 displayed blue-green colonies that were dense and fluffy, with hyphae appearing white or grayish green (Figure 1E). In microscopic observations, strain 201 showed solitary characteristics: the hyphae have septa, the conidiophore are colorless, there are a few of branches that are sharp and angular, and many circular contents can be seen inside the hyphae. Microconidia are colorless, elliptical, ovoid, and columnar, with a wide center and gradually narrowing at both ends. Meanwhile, macroconidia are achromatic, sickle shaped, slender, with 0–5 septa and mostly 2–3 septa (Figure 2A). In contrast, strain 202 exhibited a complete spore-producing structure with a double layer. Sporangia resembled broom-like branching structures attached in chain-like formations. These sporangia appeared spherical or elliptical in shape and as short columns. Conidia are spherical or nearly spherical in shape, with rough walls and small spines (Figure 2B). Based on these morphological characteristics, it was initially determined that isolated strain 201 belonged to the genus *Fusarium* while strain 202 corresponded to *Aspergillus* species. Subsequently, phylogenetic analysis confirmed the identification of these fungi.

#### Phylogenetic analysis

**Figure 3:**
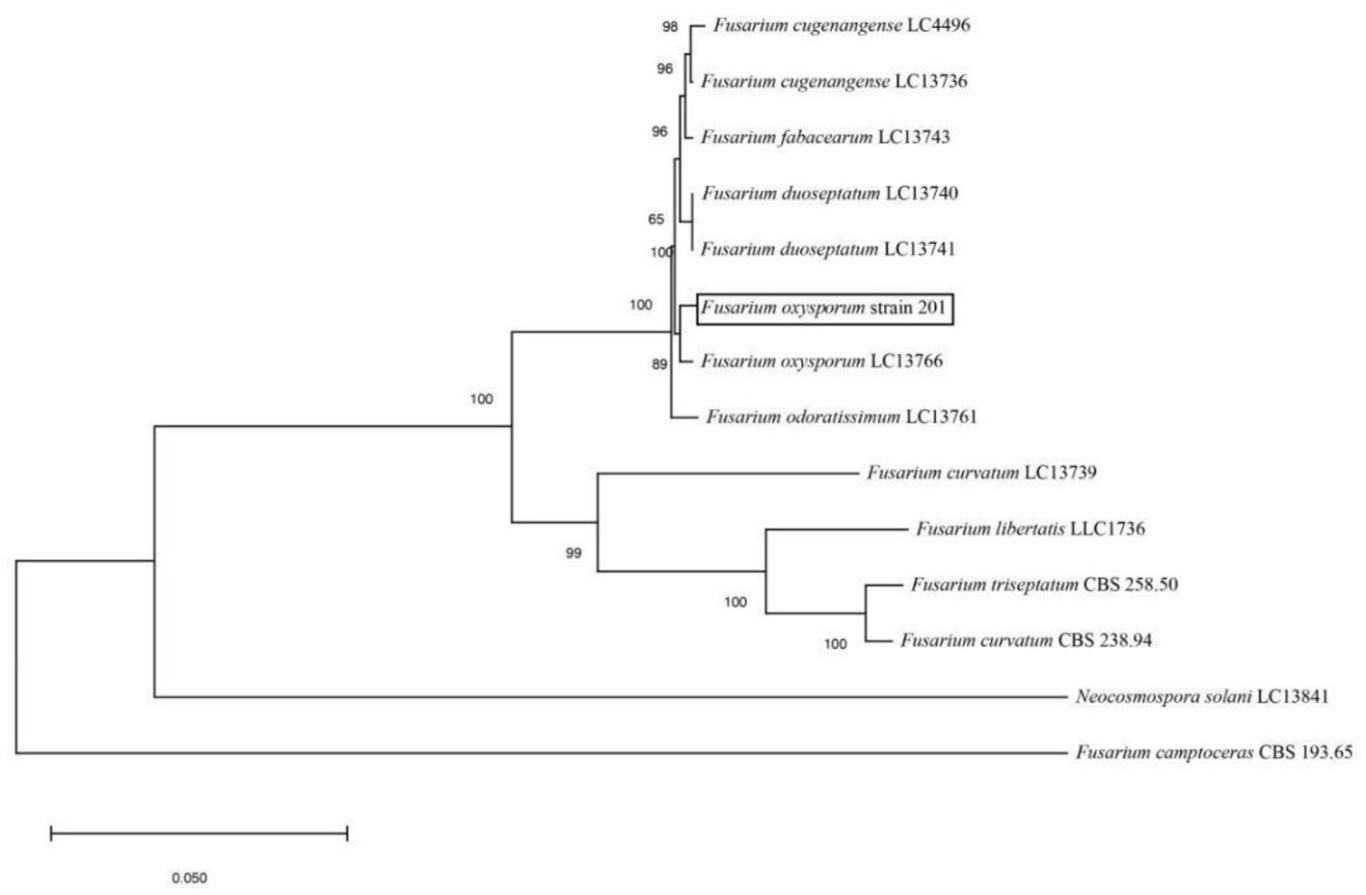
Phylogenetic analysis of concatenated sequences of Tef-1, RPB1, and RPB2 from this study and reference sequences of *Fusarium* spp. Specimens using the maximum likelihood method (1,000 bootstrap iterations).

**Figure 4:**
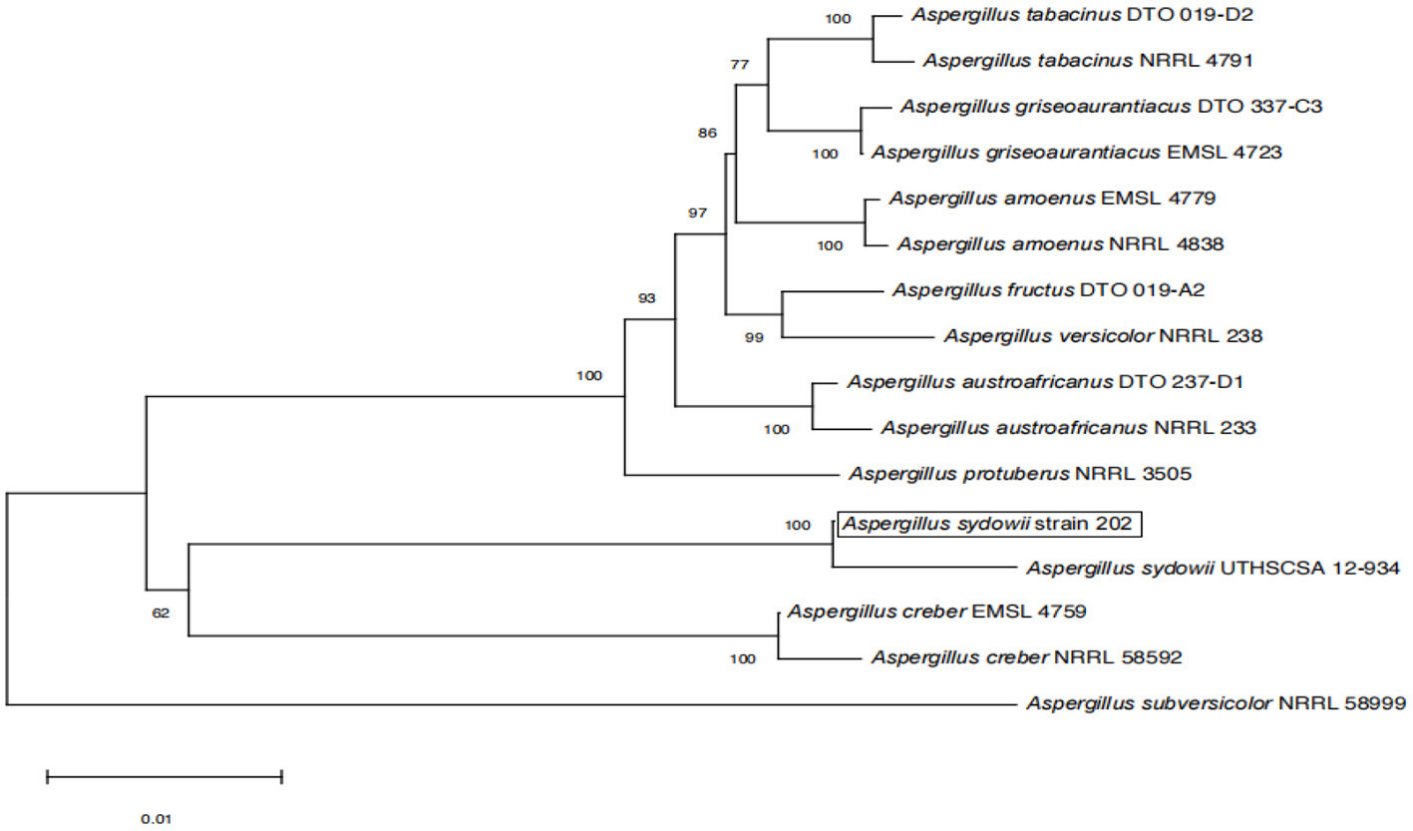
Phylogenetic analysis of concatenated sequences of BenA, Cam, RPB2, Mcm7, and Tsr1 from this study and references sequences of *Aspergillus* spp. Specimens using the maximum likelihood method (1,000 bootstrap iterations).

The amplified sequences obtained from the two isolated fungal strains in our work were deposited into the GenBank (Tables 2, 3). The basic local alignment search tool (BLAST), were used to assign strains 201 and 202 to belong to the *Fusarium* species complex and *Aspergillus* species complex, respectively. Furthermore, fungal bioassay was performed through phylogenetic analysis. In our work, two phylogenetic trees were constructed for *Fusarium* and *Aspergillus* species complex. Consequently, the maximum likelihood (ML) analysis-generated phylogenetic trees are presented here. The phylogenetic tree successfully established monophyletic branches for the two fungal isolates (strains 201 and 202) evaluated in this study. Based on this tree, we can see that strain 201 and *F. oxysporum* as well as strain 202 and *A. sydowii* were on the same branches. Both have a high statistical support rate (100% BS; Figures 3, 4).

**Table 2:** Details of the *Fusarium* species complex sequences used in the molecular phylogenetic analysis.

**Fungal taxa**	**Strain/isolate**	**GenBank accession number**		
		**Tef-1**	**RPB1**	**RPB2**
*Fusarium*	CBS 258.50	MH484964.1	MW928820.1	MH484873.1
*Fusarium*	CBS 193.65	MN170450.1	MW928800.1	MN170383.1
*F. curvatum*	CBS 238.94	MH484984.1	MW928804.1	MH484893.1
*F. cugenangense*	LC4496	MW594324	MW024599	MW474557
*F. cugenangense*	LC13736	MW594321	MW024596	MW474554
*F. curvatum*	LC13739	MW594325	MW024600	MW474558
*F. duoseptatum*	LC13740	MW594326	MW024601	MW474559
*F. duoseptatum*	LC13741	MW594327	MW024602	MW474560
*F. fabacearum*	LC13743	MW594329	MW024604	MW474562
*F. nirenbergiae*	LC13760	MW594346	MW024621	MW474579
*F. odoratissimum*	LC13761	MW594348	MW024623	MW474581
*F. oxysporum*	LC13766	MW594353	MW024628	MW474586
*F. libertatis*	LLC1736	OP487222.1	OP486375.1	OP486790.1
*N. solani*	LC13841	MW620188	MW024757	MW474713

**Table 3:** Details of the *Aspergillus* species complex sequences used in the molecular phylogenetic analysis.

**Fungal taxa**	**Strain/isolate**	**GenBank accession number**				
		**BenA**	**CaM**	**RPB2**	**Mcm7**	**Tsr1**
*A. versicolor*	NRRL 238	LC589363	EF652354	EF652178	JN854079	JN853911
*A. versicolor*	DTO 019-A2	ON807694	ON807829	ON808133	ON807971	ON808262
*A. versicolor*	NRRL 4838	EF652304	EF652392	EF652216	JN854074	JN853907
*A. versicolor*	EMSL 4779	ON807705	ON807840	ON808143	ON807985	ON808274
*A. versicolor*	NRRL 233	JN853963	JN854025	JN853814	JN854086	JN853916
*A. versicolor*	DTO 237-D1	ON807716	ON807852	ON808155	ON807998	OP688454
*A. versicolor*	NRRL 3505	EF652284	EF652372	EF652196	JN854088	LC004923
*A. versicolor*	DTO 337-C3	ON807682	ON807818	ON808122	ON807959	ON808251
*A. versicolor*	EMSL 4723	ON807683	ON807819	ON808123	ON807960	ON808252
*A. versicolor*	NRRL 4791	EF652302	EF652390	EF652214	JN854084	JN853922
*A. versicolor*	DTO 019-D2	ON807675	ON807811	ON808115	ON807951	ON808243
*A. sydowii*	CGMCC 3.06723	ON807742	ON807878	ON808178	ON808035	ON808308
*A. sydowii*	UTHSCSA 12-934	LN898879	LN898802	LN898956	ON808032	ON808305
*A. subversicolor*	NRRL 58999	JN853970	JN854010	JN853799	JN854069	JN853857
*A. creber*	EMSL 4759	ON807791	ON807928	ON808221	ON808090	ON808362
*A. creber*	NRRL 58592	JN853980	JN854043	JN853832	JQ301890	JN853887

#### Pathogenicity test

**Figure 5:**
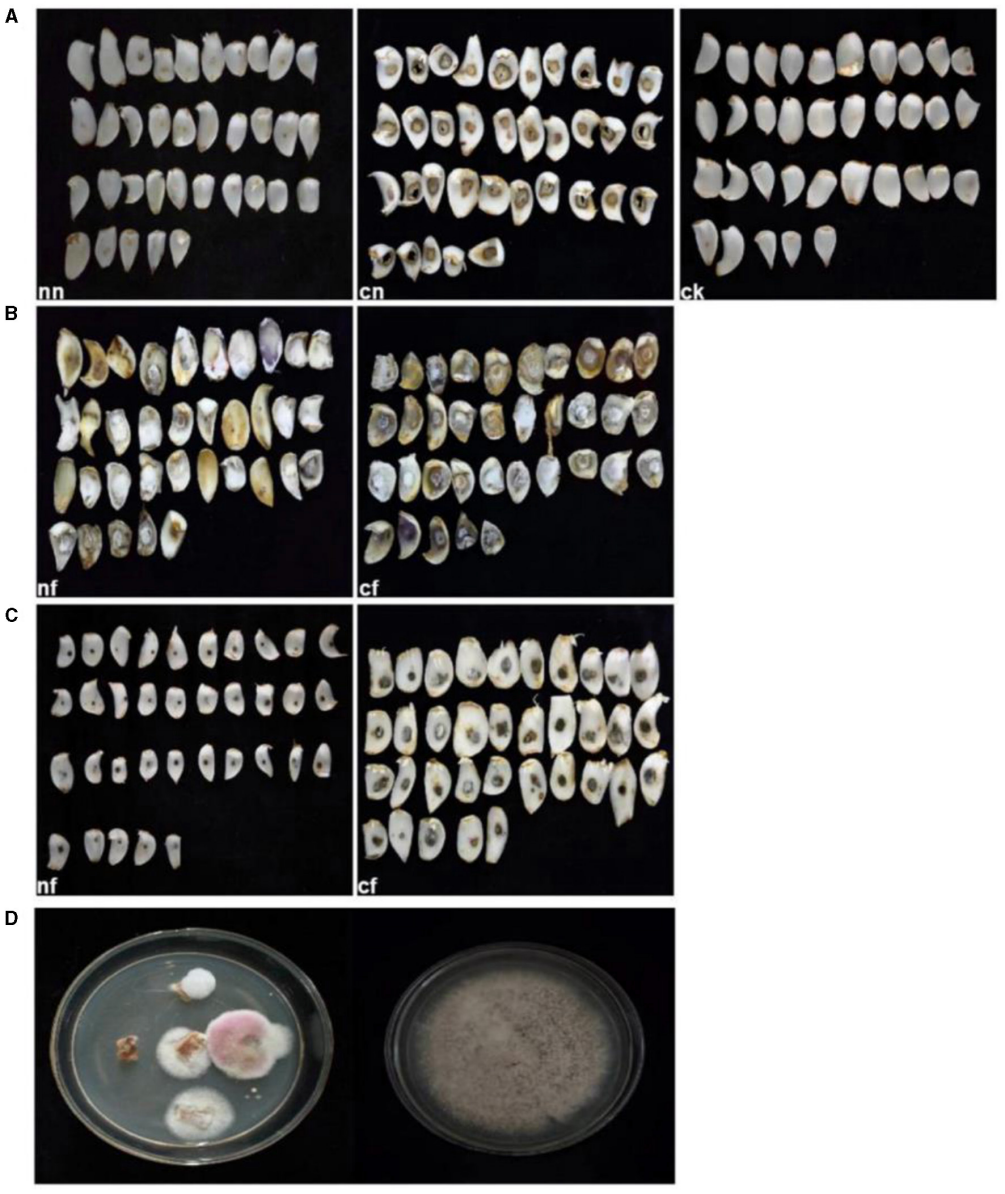
Pathogenicity test results of lily bulbs. **(A)** Represents the control group; **(B)** represents strain 201; **(C)** represents strain 202. **(D)** Pathogenicity test bulbs were reisolated and purified. **ck**, control group (fresh and undamaged bulb); **nn**, uninjured blank group (blank medium was placed in uninjured bulb slices); **cn**, stab blank group (blank medium was placed at the stab); **nf**, harmless fungus group (culture medium with the fungus was placed in the harmless bulb); **cf**, puncture fungus group (fungal culture medium placed at the puncture site).

After 15 days of inoculation, typical symptoms of BRD became visible on the bulbs. In contrast, the control group and strain 202 group remained asymptomatic (Figures 5A, C). Especially for the strain 202, there was a slight decay in the pricked area, where the fungal cake was placed in the cf group^1^. We speculated that this rot may be caused by prick injury rather than strain 202 (Figure 5C). The inoculated group pathogens of strain 201, whether injured or not, exhibited severe external damage characterized by the diseased spots of dark brown and brown lesions during infection. Surrounded by hyphae, these spots gradually expanded to form round or irregular shapes and eventually developed a rotten appearance with a visible layer of brown mold (Figure 5B). Thirty-five samples were used for the pathogenicity test, which is equivalent to 35 replicates and they consistently yielded similar results. To confirm the main pathogen, the cultures were isolated again from rot bulbs (Figure 5D) and identified to be *F. oxysporum* using the aforementioned morphological and molecular methods.

Based on the aforementioned findings, strain 201 is identified as *F. oxysporum*, while strain 202 is classified as *A. sydowii*. Because strain 201 can cause lily bulb rot and strain 202 cannot, simultaneously strain 201 can be obtained through re-isolation according to the pathogenicity test. Consequently, it can be inferred that *F. oxysporum* serves as the primary pathogenic fungi responsible for BRD in Lanzhou lily. Henceforth, the subsequent research was exclusively focused on *F. oxysporum*.

#### Influence of different culture media on mycelium growth and spore yield

**Figure 6:**
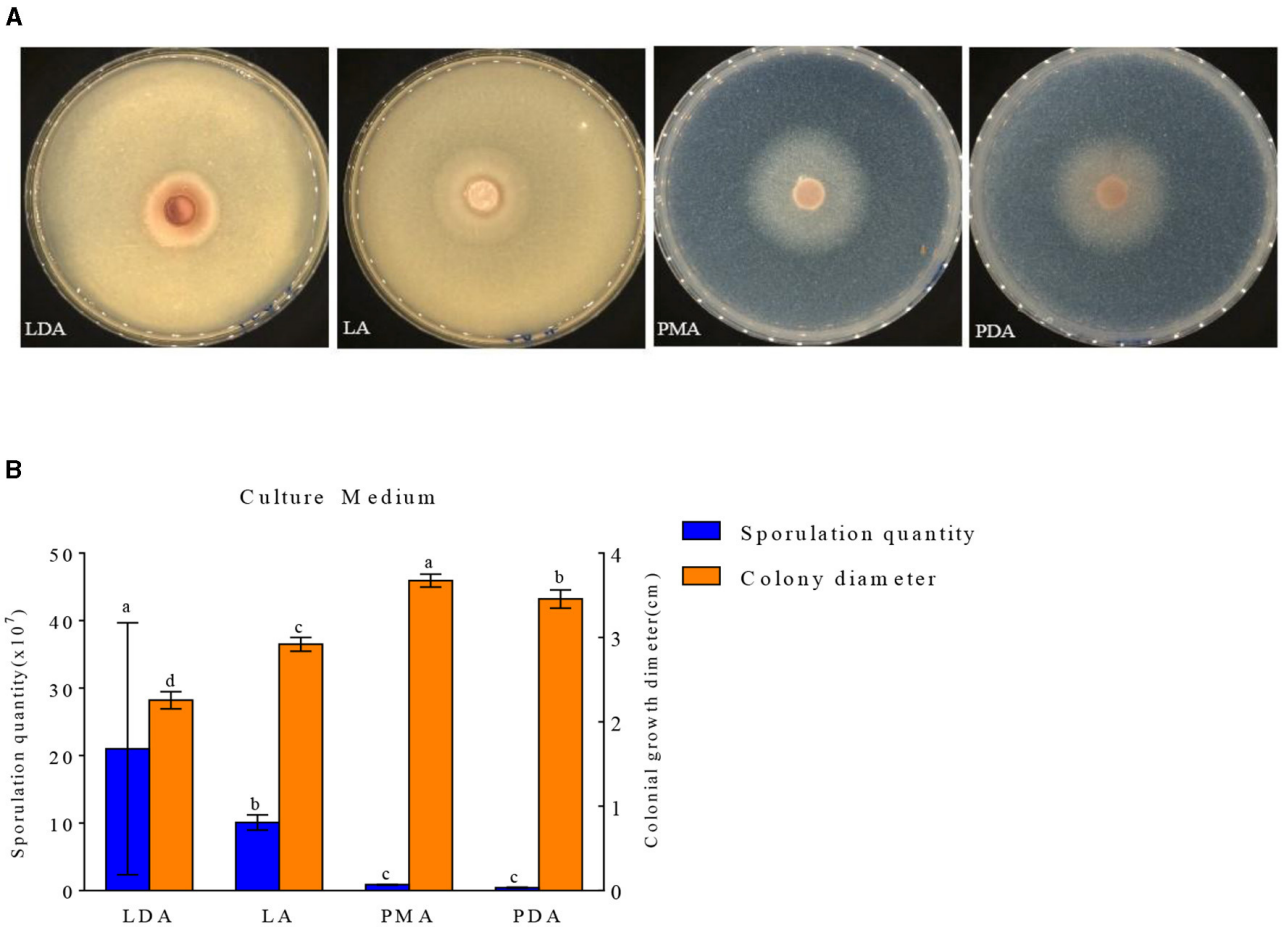
Effect of culture media on the mycelia growth and sporulation of strain 201. **(A)** The growth state of strain 201 on different media; **(B)** Histogram of the effect of different media on the mycelial growth and spore production of strain 201. Different letters indicate significant differences between groups.

The growth of mycelial growth and spore yield of *F. oxysporum* were significantly influenced by different media (Figures 6A, B). *F. oxysporum* can grow on all test media. The PMA medium and LDA medium are suitable culture media for *F. oxysporum*. It was observed that the colony diameter reached 3.68 cm after 3 days of cultivation on the PMA medium, and the spore yield was measured as 2.10 × 10^7^ cfu/mL after a duration of 7 days on the LDA medium. Secondly, LA medium showed a significant decrease in mycelial growth and an increase in spore production compared to PDA medium, but both PMA and PDA media have a cholesterol-lowering effect on the spore yield of *F. oxysporum* (Table 4). In conclusion, both PMA and LDA media demonstrate suitability for cultivating pathogenic fungi.

**Table 4:** Effect of culture media on the mycelia growth and sporulation of strain 201.

**Medium type**	**Spore yield (× 10^7^ cfu/mL)**	**Colony diameter (cm)**
LDA	21.02 ± 17.61a	2.25 ± 0.08d
LA	10.09 ± 1.06b	2.92 ± 0.06c
PMA	0.88 ± 0.06c	3.67 ± 0.06a
PDA	0.46 ± 0.05c	3.45 ± 0.09b

Different letters indicate significant differences between groups (*P* < 0.05).

#### Use of different carbon and nitrogen sources

**Figure 7:**
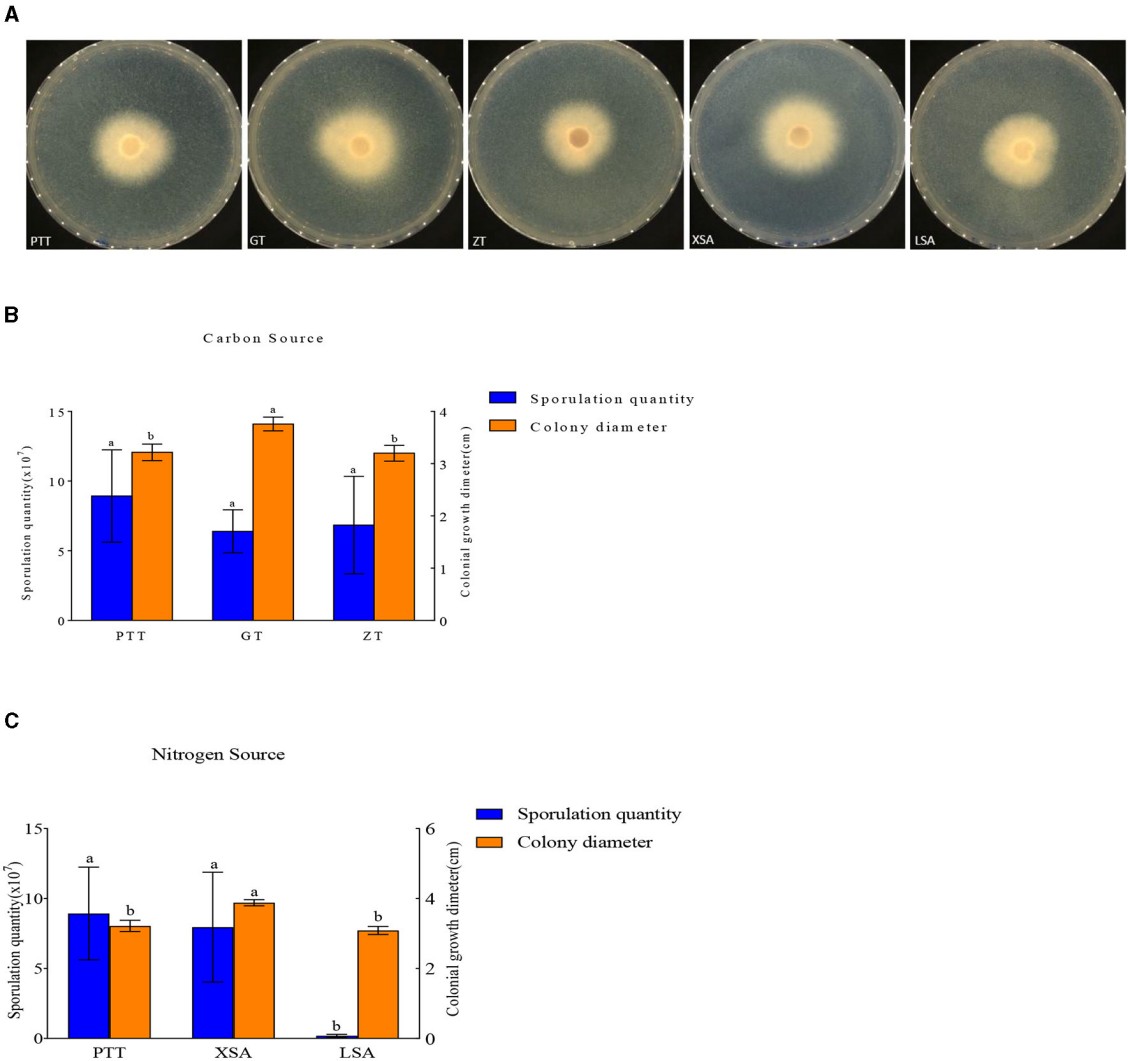
Effect of carbon and nitrogen source on the mycelia growth and sporulation of strain 201. **(A)** The growth status of strain 201 on different carbon and nitrogen sources; **(B, C)** Histogram of the effect of carbon and nitrogen sources on the mycelial growth and spore production of strain 201. Different letters indicate significant differences between groups.

The used carbon and nitrogen sources in all the experiments supported mycelium growth (Figure 7). Compared with the control medium (PTT), *F. oxysporum* exhibited the fastest mycelium growth and produced the highest spores on a culture medium with ammonium nitrate (XSA) as the nitrogen source, with a colony diameter of 3.88 cm after 3 days and the spore production of 8.00 × 10^7^ cfu/mL after 7 days. On the other hand, the fastest growth and highest spore production of *F. oxysporum* were observed on the culture medium using fructose (GT) as the carbon source, in which the colony diameter was 3.76 cm after 3 days, and the spore production was 6.4 × 10^7^ cfu/mL after 7 days, with statistically significant differences observed (Table 5). Overall, the conditions of XSA as the nitrogen source and GT as the carbon source are more favorable for the growth of *F. oxysporum*.

**Table 5:** Effect of carbon and nitrogen sources on the mycelia growth and sporulation of strain 201.

**Medium type**		**Spore yield (× 10^7^ cfu/mL)**	**Colony diameter (cm)**
Control	PTT	8.90 ± 3.13a	3.21 ± 0.13b
	GT	6.40 ± 1.46a	3.76 ± 0.11a
Control source	ZT	0.68 ± 3.30a	3.20 ± 0.13b
	XSA	8.00 ± 3.70a	3.88 ± 0.07a
Nitrogen source	LSA	0.19 ± 0.10b	3.08 ± 0.10b

Different letters indicate significant differences between groups (*P* < 0.05).

#### Effect of temperature and relative humidity

**Figure 8:**
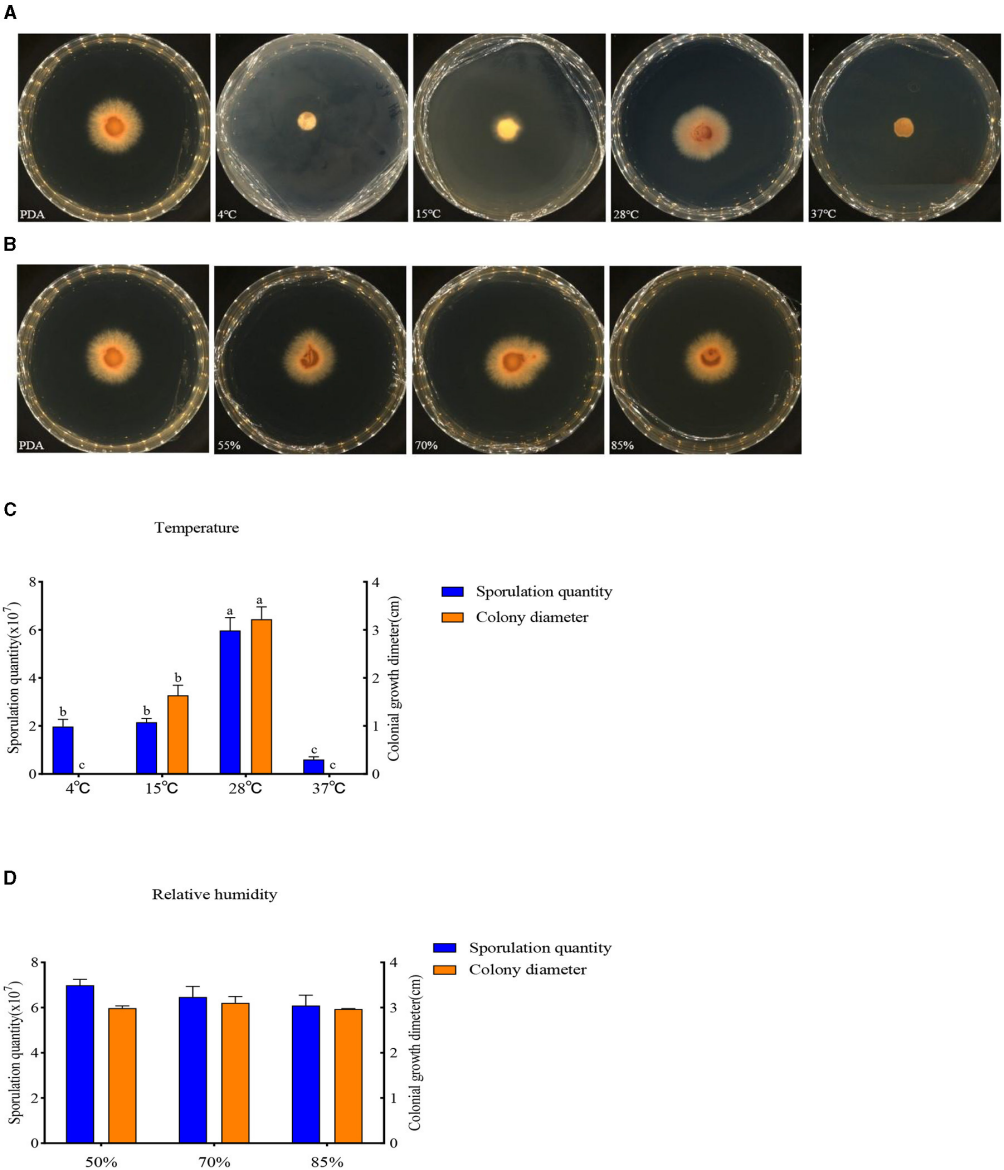
Effect of temperature and relative humidity on the mycelia growth and sporulation of strain 20. **(A, B)** The growth state of strain 201 under different temperatures and relative humidity values; **(C, D)** Histogram of the effect of temperature and relative humidity on the mycelial growth and spore production of strain 201. Different letters indicate significant differences between groups.

The buildup of *F. oxysporum* is significantly influenced by temperature especially (Figure 8). At temperatures of 4, 15, 28, and 37°C, mycelial growth and spore production were observed. At 4 and 37°C, the mycelium stops growing and the spore production decreases; the optimal temperature for the growth of *F. oxysporum* is 28°C, with the fastest mycelium growth and maximum spore production, followed by 15°C. *F. oxysporum* grows at intervals of 15% in the relative humidity range of 55–85%. Notably, RH did not have an obvious impact on the buildup of *F. oxysporum*. However, the spore production was most mutative when the relative humidity was 55% (Table 6). On the whole, the temperature of 28°C and relative humidity of 55% were the best conditions for the growth of mycelium and the spore production of *F. oxysporum*.

**Table 6:** Effect of temperature and relative humidity on the mycelia growth and sporulation of strain 201.

**Type**	**Spore yield (**×**10**^**7**^ **cfu/mL)**		**Colony diameter (cm)**
Temperature	4°C	1.83 ± 0.49b	0.00c
	15°C	2.13 ± 0.35b	1.64 ± 0.21b
	28°C	6.24 ± 0.87a	3.22 ± 0.26a
	37°C	0.51 ± 0.18c	0.00c
Relative humidity	55%	6.78 ± 0.50	2.98 ± 0.05
	70%	6.16 ± 0.54	3.10 ± 0.14
	85%	6.10 ± 0.68	2.96 ± 0.01

Different letters indicate significant differences between groups (*P* < 0.05).

#### Influence of pH and photoperiod on mycelium growth and spore yield

**Figure 9:**
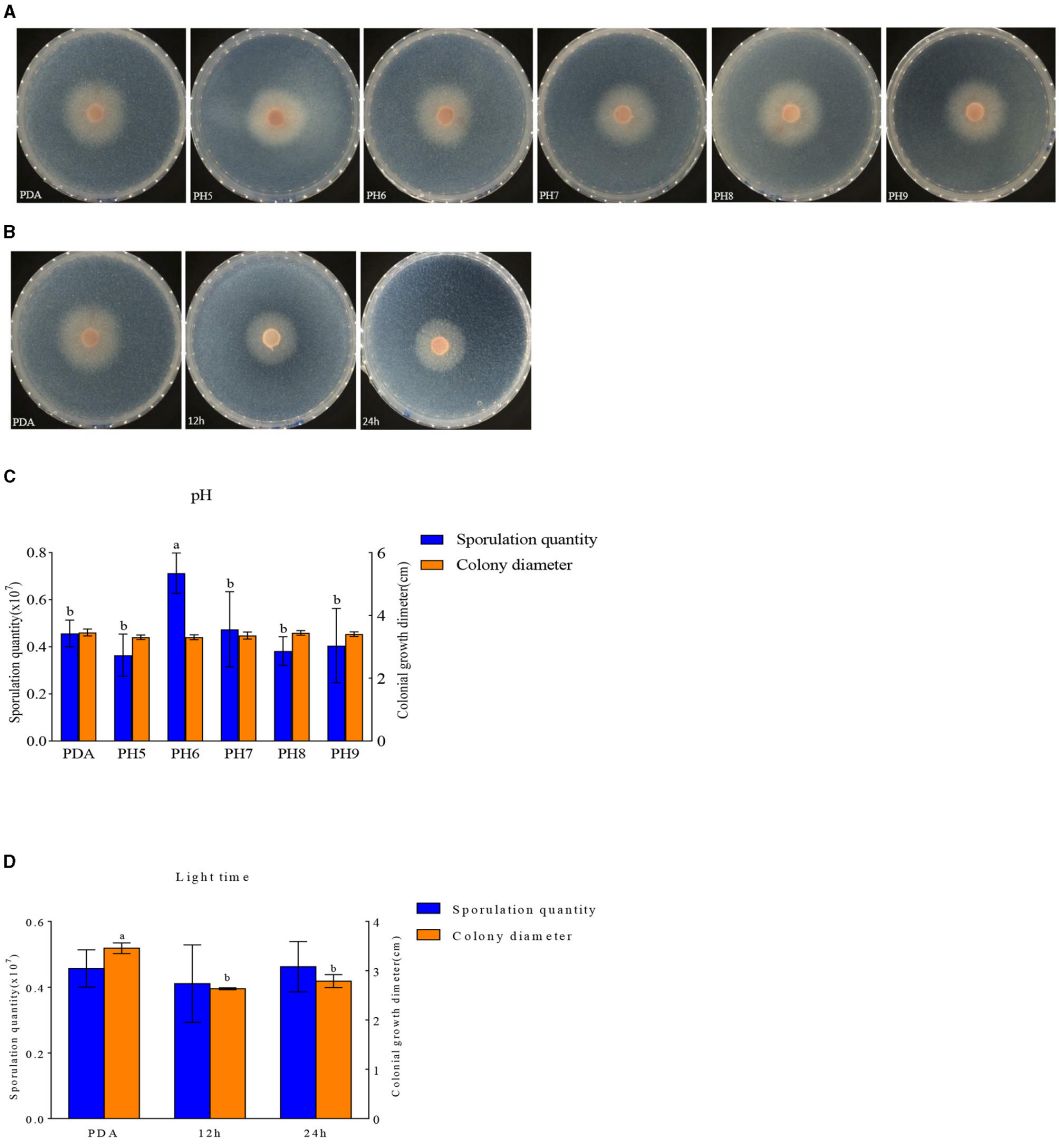
Effect of pH and light treatment on the mycelia growth and sporulation of strain 201. **(A, B)** Growth state of strain 201 under different pH and light; **(C, D)** Histogram of the effect of pH and light on the mycelial growth and spore production of strain 201. Different letters indicate significant differences between groups.

*F. oxysporum* exhibits the ability to thrive in the pH range of 5–9 (Figures 9A, C). Other pH values have no effect on the growth of *F. oxysporum*, but the spore production of *F. oxysporum* is highest reaching 0.71 × 10^7^ cfu/mL when pH is 6 (Table 7). Photoperiod also plays an important role on the growth of *F. oxysporum* (Figures 9B, D). It was observed that the light conditions favorable for *F. oxysporum* growth and spore yield of *F. oxysporum* are completely dark, followed by the conditions of complete light, and finally light/dark for 12 h. Based on the comprehensive analysis, the pH of 6 and completely dark were the most suitable factors for the growth of *F. oxysporum*.

**Table 7:** Effect of pH and light treatment on the mycelia growth and sporulation of strain 201.

**Type**	**Spore yield (**×**10**^**7**^ **cfu/mL)**		**Colony diameter (cm)**
pH	PDA	0.46 ± 0.05b	3.46 ± 0.09
	5	0.36 ± 0.08b	3.33 ± 0.02
	6	0.71 ± 0.08a	3.30 ± 0.06
	7	0.47 ± 0.15b	3.36 ± 0.09
	8	0.38 ± 0.06b	3.44 ± 0.06
	9	0.41 ± 0.11b	3.40 ± 0.06
Light time	PDA	0.46 ± 0.05	3.46 ± 0.09a
	12 h	0.41 ± 0.11	2.63 ± 0.02b
	24 h	0.46 ± 0.07	2.79 ± 0.12b

Different letters indicate significant differences between groups (*P* < 0.05).

#### Fungicide assays

**Figure 10:**
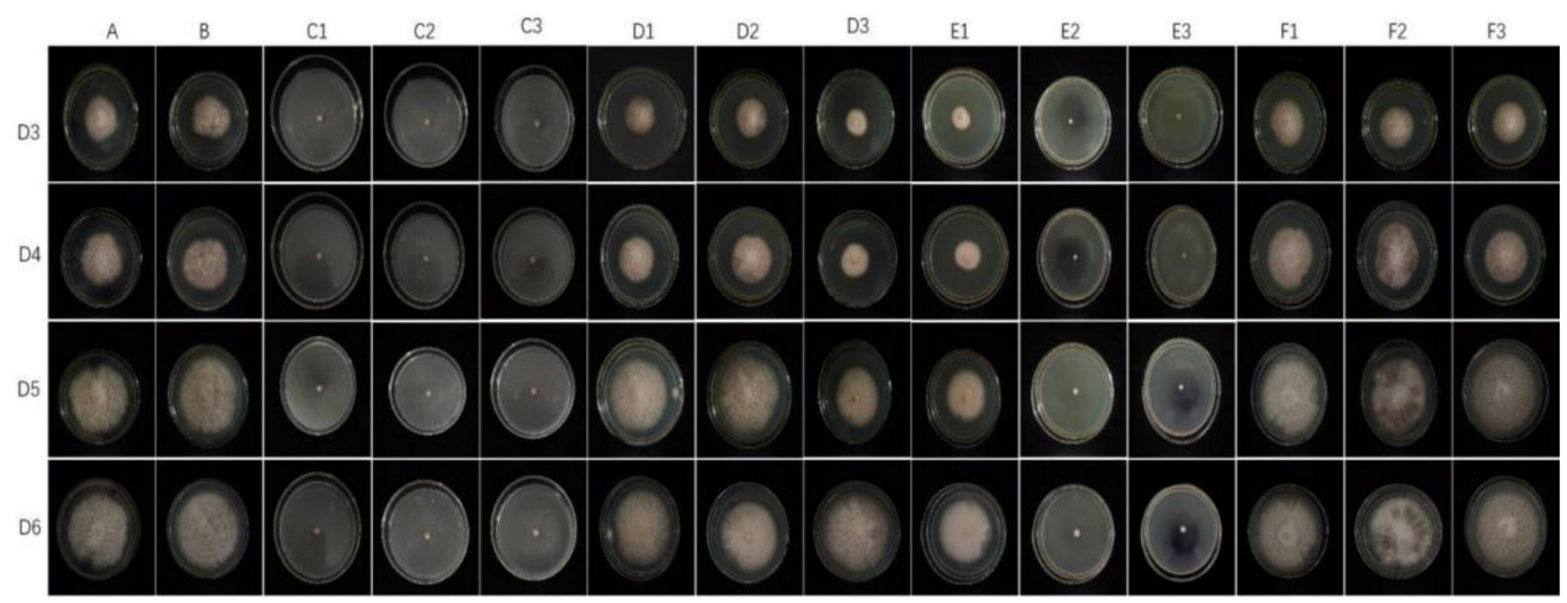
Effect of different antifungal agents on strain 201. Mycelial growth of strain 201 on PDA plates incubated for 3–6 days in the control group (A: blank medium, B: medium+water), positive control group (C: carbendazim, C1: 0.14 μL/mL, C2: 0.17 μL/mL, C3: 0.2 μL/mL), presence of different concentrations of angelica essential oil (D1: 0.14 μL/mL, D2: 0.17 μL/mL, D3: 0.2 μL/mL), cinnamon essential oil (E1: 0.14 μL/mL, E2: 0.17 μL/mL, E3: 0.2 μL/mL), and tea tree essential oil (F1: 0.14 μL/mL, F2: 0.17 μL/mL, F3: 0.2 μL/mL).

The representative isolate *F. oxysporum* exhibited varying sensitivities to the three plant essential oils (Figure 10). Among the tested essential oils, cinnamon essential oil demonstrated the highest inhibition rate, exhibiting a comparable antifungal effect to carbendazim within a specific range. Next was angelica essential oil, which also displayed suppressive effects against *F. oxysporum*. In contrast, when compared to cinnamon essential oil and *Angelica sinensis* essential oil, tea tree essential oil exhibits the weakest inhibitory effect on *F. oxysporum*. These findings indicate that *F. oxysporum* is most susceptible to cinnamon essential oil and least susceptible to tea tree essential oil (Table 8).

**Table 8:** Effect of the treatment with different plant essential oils on the mycelia growth of strain 201 (cm).

	**D3**	**D4**	**D5**	**D6**
A	4.67 ± 0.03	5.82 ± 0.06	7.52 ± 0.06	8.06 ± 0.05
B	5.18 ± 0.02^a^	5.85 ± 0.24	7.40 ± 0.08	8.28 ± 0.06
C1	0.60^ab^	0.60^ab^	0.60^ab^	0.60^ab^
C2	0.60^ab^	0.60^ab^	0.60^ab^	0.60^ab^
C3	0.60^ab^	0.60^ab^	0.60^ab^	0.60^ab^
D1	4.02 ± 0.06^a^	5.23 ± 0.22^a^	6.65 ± 0.08^a^	7.52 ± 0.05^a^
D2	3.02 ± 0.07^ac^	3.83 ± 0.10^ac^	5.30 ± 0.01^ac^	6.02 ± 0.05^ac^
D3	4.23 ± 0.02^ac^	5.38 ± 0.02^a^	6.90 ± 0.04^ac^	7.90 ± 0.04^c^
E1	2.60 ± 0.08^a^	3.50 ± 0.12^ac^	4.78 ± 0.18^a^	6.05 ± 0.16^a^
E2	0.60^ad^	0.60^ad^	0.60^ad^	0.61 ± 0.11^ad^
E3	0.60^ad^	0.60^ad^	0.60^ad^	0.60^ad^
F1	5.13 ± 0.14^a^	6.40 ± 0.08^a^	7.93 ± 0.14^a^	8.32 ± 0.07
F2	4.89 ± 0.06^a^	6.19 ± 0.05^ae^	6.85 ± 0.04^ae^	8.05 ± 0.16
F3	4.60 ± 0.05^d^	5.89 ± 0.04^d^	7.62 ± 0.06^d^	8.39 ± 0.05^ae^

^a^Represents the comparison between each group and Group A.

^b^Represents the comparison between C1, C2, and C3.

^c^Represents the comparison between D1, D2, and D3.

^d^Represents the comparison between E1, E2, and E3.

^e^Represents the comparison between F1, F2, and F3, P < 0.05.

## Discussion

In this study, we isolated two strains of fungi from the rotten bulbs of Lanzhou lilies, namely, *F. oxysporum* and *A. sydowii*. *F. oxysporum* was identified as the main causative factor of BRD in Lanzhou lily based on comprehensive morphological and molecular characterization along with rigorous pathogenicity tests. Previous research (Bian et al., 2016) has reported the isolation of pathogenic fungi such as *F. oxysporum* from Lanzhou lily planted in Lintao County, Gansu Province. *F. oxysporum* could also cause scale rot of Lanzhou lily in the process of air culture, and the incidence rate was 95% (Hu et al., 2022), which is consistent with our findings regarding the isolation of pathogenic fungi.

*A. sydowii*, a species of *Aspergillus*, is widely distributed in nature due to its strong survival ability (Yu, 2015). Based on existing literature, research on *A. sydowii* has mostly focused on marine *A. sydowii* and tobacco products, as marine *A. sydowii* can produce various bioactive secondary metabolites (Sun, 2022). In addition, researchers have isolated *A. sydowii* in tobacco products (Huang et al., 2022; Zhu et al., 2023). For our study, *A. sydowii* was isolated for the first time. To our knowledge, this is one of the few reports on the isolation of *A. sydowii* from Lanzhou lily with BRD.

Furthermore, we characterized the influence of various carbon and nitrogen sources on the mycelium growth and spore production of *F. oxysporum*. Studies on biological characteristics have shown that the growth of *F. oxysporum* is the fastest on PMA medium, and the spore yield is the highest on the LDA medium. This indicates that the nutrition of maltose and lily bulbs themselves are beneficial for the reproduction of *F. oxysporum*, which can infect lily bulbs and cause rot disease. In our study, ammonium nitrate as the nitrogen resource can promote mycelium growth and spore production of *F. oxysporum*. For this reason, we recommend that careful consideration should be given to the application of ammonium nitrate as a fertilizer in the fields of Lanzhou lily affected by *F. oxysporum* in China. In addition, we found that the tested carbon sources supported mycelium growth and spore production. Yet, compared to other carbon resources, the pathogen exhibits higher speed of growth and spore production on fructose. Other reports have shown that sucrose is one of the best carbon sources for producing enrofloxacin in *F. oxysporum* isolates (Cao et al., 2018; Ya et al., 2018), but both fructose and sucrose can alter the biosynthesis of fungal toxins and affect the quality of lilies. In the future research, it will be vital to study how carbon and nitrogen resources other than NH_4_NO_3_ and fructose affect lily BRD and explore how these resources can be considered and utilized in lily cultivation and storage. At the same time, carbon and nitrogen resources supporting BRD development and fungal toxin production should be reduced, which can provide new solutions for sustainable management of lily bulb diseases and storage.

The mycelial growth of *F. oxysporum* was influenced heavily by temperature. The mycelium stops growing and the spore production decreases at 4 and 37°C. *F. oxysporum* can produce spores when the temperature is between 4 and 37°C. The optimal growth temperature range for mycelium growth is 15–28°C, but 28°C is the appropriate temperature for mycelial growth as well as spore production. This finding agrees with Bian et al.'s (2016) and Cao et al. (2018) research on the biological characteristics of *F. oxysporum*, which revealed that the optimal temperature for mycelial growth and spore production was 25°C. In addition, humidity is an important environmental factor having a certain impact on the occurrence of BRD in Lanzhou lily. In this study, a relative humidity of 55% was found to be optimal for mycelial growth and spore production of *F. oxysporum*. Therefore, we should fully consider the prevention of BRD in Lanzhou lily by varying the temperature and relative humidity during its storage.

According to reports, photoperiod as a key external environmental variable may affect the plant pathogens and their biological characteristics (Costa et al., 2021; Macioszek et al., 2021). In our present work, full darkness (no illumination) was conducive to the buildup of *F. oxysporum*. For some isolates of *F. oxysporum*, total darkness was also clearly causing hyphal growth and/or spore yield (Pan et al., 2011; Jin et al., 2019). This is different from the observation of Cao Xing, who have indicated that the light and dark environment affects its mycelial growth and conidia germination (Cao et al., 2018). Accordingly, within *Fusarium* sp., strain specificity reactions to photoperiod may extensively exist. When the pH increases between pH 5 and 9, there is no significant change in the size of the colony diameter and spore production of *F. oxysporum*. But the spore production reaches its maximum value at pH 6. Moreover, pH 6–7 is conducive to mycelial growth and spore production, with pH 6 being the optimal value. These findings agree with Zhao (2012), who found the optimal pH of 6 for the mycelial growth of *F. oxysporum*, a pathogen of green hybrid bamboo wilt disease, and also consistent with Feng's (2014) description of the optimal pH 6 for *F. oxysporum*, a pathogen of Acacia wilt disease. Consequently, it is necessary to explore the effects of ultraviolet radiation and soil pH on bulb diseases of Lanzhou lily during storage.

The utilization of natural plant ingredients with antifungal properties as potential alternatives to chemical fungicides is preferred. Consequently, we conducted an investigation on the antifungal efficacy of three plant essential oils against *F. oxysporum*. Among the three tested essential oils, cinnamon essential oil showed the best inhibition rates. The results were consistent with the research findings that cinnamon essential oil was good for inhibiting the growth of bacteria and fungi (Mao, 2018; He, 2019; Yang et al., 2021; Zhao et al., 2023). Some researchers have proven that angelica essential oil has an inhibitory effect on pathogenic bacteria (Prakash et al., 2015; Qiao et al., 2022). Our results indicated that angelica essential oil s also showed an inhibition ability against *F. oxysporum*. Similar to our research findings, several biological fungicides and their mixtures were shown to inhibit the growth of *F. oxysporum* isolates (Jiang et al., 2021). Therefore, it is necessary to conduct in-depth and systematic research in the future to determine whether a mixture of various plant essential oils can more effectively inhibit the fungal diseases of Lanzhou lily during storage.

## Data availability statement

The original contributions presented in the study are included in the article/supplementary material, further inquiries can be directed to the corresponding author.

## Author contributions

CL: Writing—original draft, Writing—review & editing. YiW: Writing—review & editing, Project administration, Supervision. LJ: Project administration, Writing—review & editing. YaW: Writing—review & editing. DL: Writing—review & editing.

## Footnotes

^1^“cf” group refers to the fungi cake with PDA culture medium were pasted on the hole as the injured inoculation,means inoculate pathogenic fungi at the puncture site (here pathogenic fungi are strain 201 and strain 202).
